# Neutropenia (even mild) and anemia are poor prognostic factors in myelodysplastic syndromes

**DOI:** 10.3389/fmed.2025.1558585

**Published:** 2025-07-11

**Authors:** Reut Hausman, Roi Gat, Noa Goldschmidt, Moshe Mittelman, Howard S. Oster

**Affiliations:** ^1^Department of Medicine, Tel Aviv Sourasky Medical Center, Tel Aviv, Israel; ^2^Department of Hematology, Tel Aviv Sourasky Medical Center, Tel Aviv, Israel; ^3^The MDS Center of Excellence, Tel Aviv Sourasky Medical Center, Tel Aviv, Israel; ^4^Faculty of Medicine, Tel Aviv University, Tel Aviv, Israel

**Keywords:** myelodysplastic syndromes, neutropenia, anemia, prognosis, mortality, leukemic transformation

## Abstract

**Background:**

Severe neutropenia (ANC < 0.8 × 10^9^/L) indicates poor MDS prognosis (IPSS-R classification). The impact of mild neutropenia is unclear.

**Methods:**

We compared baseline and outcomes (not infections) of 50 consecutive patients with neutropenia (Neutp, ANC < 1.5 × 10^9^/L) to 50 non-neutropenic (Non-Neutp).

**Results:**

Both groups were similar: Age 74.8 years; 61% males; ECOG 0/1 (91%); comorbidities. In Neutp vs. Non-Neutp patients: Hb (9.8 vs. 10.9 g/dL); WBC (2.7 vs. 7.7 × 10^9^/L); Lymphocytes (1.2 vs. 1.8 × 10^9^/L); Monocytes (0.46 vs. 0.73 × 10^9^/L); PLT (115 vs. 201 × 10^9^/L). Ferritin was higher (642 vs. 304 ng/mL, *p* = 0.002). BM dyserythropoiesis was less (50% vs. 72%, *p* = 0.04), while dysmyelopoiesis (48% vs. 26%) and blasts (3.3% vs. 1.1%, *p* < 0.001) were more common. More Neutp patients (60.0%) were classified as HR-IPSSR than Non-Neutp (12.2%, *p* < 0.001). The median OS was shorter (101 vs. 122 m, but *p* = 0.12); 18 (36%) Neutp vs. 6 (12%) Non-Neutp patients transformed to AML (*p* = 0.002), with a shorter TTL (*p* = 0.002). The median time to composite endpoint (death or leukemic transformation) was 82 vs. 114 m (*p* = 0.035). In a Cox proportional hazard model, CVD affected OS, while cytogenetics and neutropenia affected leukemic transformation and composite outcome. Lymphocytes, monocytes and platelets had no impact on outcomes. Patients with only neutropenia or only anemia (HB < 10) had a small, non-significant impact, but patients with both had a profound impact on all outcomes (composite: HR = 4.15, 95% CI [2.25–7.7], *p* < 0.001).

**Conclusion:**

Mild neutropenia, especially with anemia, is a poor prognostic factor in MDS. These patients have more BM failure and worse outcomes (OS, leukemic transformation, TTL).

## Graphical Abstract

Mild neutropenia and anemia – poor prognosis in MDS. 
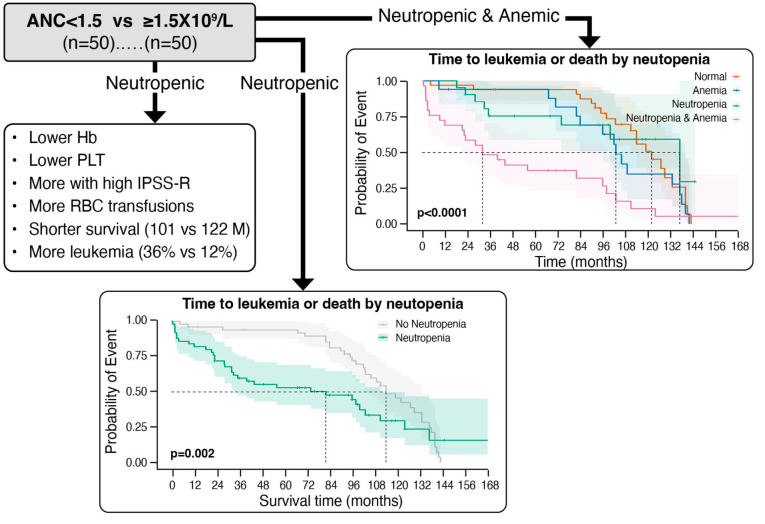

## Introduction

The myelodysplastic syndromes (MDS) are a heterogenous group of clonal myeloid neoplasms originating in hematopoietic stem cells. They are characterized by ineffective hematopoiesis resulting in dysplasia in hematopoietic cells, and are associated with peripheral blood cytopenias, especially anemia, and a propensity to leukemic transformation (1–7). The incidence of MDS increases with age and in the general population is approximately 5 cases per 100,000 people per year. The median age of onset is above the age of 70 (1–8). Patients with MDS are classified using one of several scoring systems (9–14). Most patients are assigned to the lower-risk (LR) or higher-risk (HR) groups.

Neutropenia is common in MDS (10, 15, 16). In the International Prognostic Scoring System Revised classification (IPSS-R), ANC < 0.8 × 10^9^/L was considered a prognostic factor and added 0.5 points to the score (11). It’s conceivable to assume that neutropenia, especially severe, might predispose to infections. However, there is a paucity of data on the prognostic role of neutropenia, especially mild, as a single factor, its association with other variables and prediction of disease outcomes. Answering these questions was the aim of this study. As such, we examined the impact of even mild neutropenia (ANC < 1.5 × 10^9^/L) on MDS patient outcomes. Moreover, because anemia is especially common in MDS, we examined the impact of neutropenia alone, anemia alone and the combination of both on these outcomes.

## Patients and methods

### Database

The database of the MDS Center of Excellence and MDS patient cohort at Tel Aviv Sourasky Medical Center (TASMC) between the years 2011–2021 was used as the source of data.

### Patients

The inclusion criteria were: (1) MDS diagnosis based on bone marrow (BM) examination, as well as other acceptable international diagnostic criteria (3, 17, 18). (2) Age 18–90 years. (3) Having all parameters of routine blood count (CBC) and lab chemistry at presentation along with follow up data. The number of patients was 100 in total, 50 in each arm. Data were retrieved from consecutive patients who met the inclusion criteria, and were collected until we reached the number 50 in each group. We excluded patients whose MDS diagnosis was questionable (inconclusive, suspected, tentative, to rule out, most probably, idiopathic cytopenia, clonal cytopenia, no BM report available), patients whose lab data were from later period than the MDS diagnosis time and patients who were lost to follow up.

### Study design

The study was retrospective. The patient cohort was divided into two arms: (a) Neutropenic MDS patients (Neutp, absolute neutrophil count, ANC < 1.5 × 10^9^/L). (b) Non-neutropenic MDS patients (Non-Neutp, ANC ≥ 1.5 × 10^9^/L). The patient electronic medical records (EMR) were reviewed and baseline epidemiological/demographic, clinical and lab data were collected and compared between both groups, focusing on associations with various variables. Also, clinical data during follow up, treatments, course and outcomes, especially the incidence of leukemic transformation, time to leukemia (TTL) and overall survival (OS) were analyzed and compared. Intervals were calculated from the MDS diagnosis date, defined as the date of the BM examination. OS was defined as the time interval from MDS diagnosis until death or censored at time of last patient follow-up. TTL was calculated from MDS diagnosis to date of diagnosis of acute myeloid leukemia (AML). Finally, we applied Cox proportional hazard models to study the impact of five variables (age, sex, cardiovascular disease, cytogenetics – favorable vs. intermediate/poor—and neutropenia) as risk factors for three outcomes (mortality, leukemic transformation, and the composite of both, whichever comes first) and estimated the hazard ratios (HRs) and significance (*p*) of each variable.

With the realization that there may be confounding factors, we performed an additional analysis of the data where we included monocytes, lymphocytes, platelets, hemoglobin and BM blasts. Regarding hemoglobin, we examined patients in 4 categories: (1) neither neutropenia nor anemia (HB < 10), (2) neutropenia only, (3) anemia only, and (4) both neutropenia and anemia.

Infections were not studied due to the paucity of information in the MDS clinic patient charts. In Israel most MDS patients are followed and treated in tertiary hospitals and in the MDS center, but when they have an infection, they are treated either at home, in the community or in another (local) hospital. Only a few, mainly with the more serious infections, are admitted to TASMC. Also, infections are reported often in the text and not necessarily listed in the list of diagnoses. Thus, in order to avoid inaccurate analysis, we did not address infections in this study.

## Statistical methods

Patient characteristics were compared between these two groups using appropriate statistical tests based on the type of variable. Normality of continuous variables was assessed using the Anderson–Darling test. Continuous variables were summarized using the median and interquartile range (IQR), The comparisons between groups were performed using the Mann–Whitney U test, as variable do not follow normal distribution.

Categorical variables were summarized using counts and percentages. Fisher’s exact test was used to compare categorical variables between the two groups.

Survival analysis was performed using the Kaplan–Meier method to estimate survival functions for time to mortality, time to leukemia transformation, and a composite outcome of the time to the first of these events. The log-rank test was applied to compare survival distributions between groups.

The significance level was defined as two-sided (*p* < 0.05). All statistical analyses were carried out using R, version 4.4.1 (R Foundation for Statistical Computing, Vienna, Austria).

## Results

The two MDS patient populations were similar to each other in baseline characteristics (Table 1). For the Neutp and Non-Neutp groups, respectively, the mean age was 76.5 and 78.5 years, and 42% and 36% were females. Most (94% and 88%) had ECOG performance status 0 or 1. Cardiovascular disease (CVD, 44% and 54%), diabetes mellitus (32% and 32%), hyperlipidemia (14% and 6%), and hypothyroidism (20% and 14%) were the common comorbidities. Except for anticoagulants (22% vs. 40%, *p* = 0.08), the use of medications was similar.

**TABLE 1:** Patient characteristics—neutropenic vs. non-neutropenic MDS patients.

Parameter		All MDS *n* = 100	Neutropenic (*n* = 50)	Non-neutropenic (*n* = 50)	*P*-value
Age years (median and IQR)		78 (69.00, 83.25)	76.5 (66.25, 82.50)	78.5 (72.00, 84.00)	0.348
Sex: females *n* (%)		39 (39%)	21 (42%)	18 (36%)	0.682
ECOG *n* (%)	0	56 (56%)	26 (**52%**)	30 (60%)	0.321
	1	35 (35%)	21 (42%)	14 (28%)	
	2+	9 (9%)	3 (6%)	6 (12%)	
Comorbidities *n* (%)	CVD	49 (49%)	22 (44%)	27 (54%)	0.424
	Diabetes	32 (32%)	16 (32%)	16 (32%)	1.000
	Hyperlipidemia	10 (10%)	7 (14%)	3 (6%)	0.310
	Non-hematological malignancy	16 (16%)	7 (14%)	9 (18%)	0.786
	Hypothyroidism	17 (17%)	10 (20%)	7 (14%)	0.595
Medications *n* (%)	Anti-diabetics	26 (26%)	13 (26%)	13 (26%)	1.000
	Anticoagulants	31 (31%)	11 (22%)	20 (40%)	0.083
	Steroids	8 (8%)	3 (6%)	5 (10%)	0.715
	Lipid lowering	27 (27%)	12 (24%)	15 (30%)	0.653
	Thyroid agents	13 (13%)	9 (18%)	4 (8%)	0.234
	PPI	16 (16%)	11 (22%)	5 (10%)	0.171

Neutropenic: ANC < 1.5 × 10^9^/L; non-neutropenic: ANC ≥ 1.5 × 10^9^/L. CVD, cardiovascular disease; PPI, proton pump inhibitor.

Baseline hematologic indices other than ANC were significantly lower in the Neutp than in Non-Neutp patients (Table 2): Hemoglobin (Hb, 9.7 vs. 10.6 g/dL, *p* = 0.015); White blood cells (WBC, 2.6 × 10^9^/L vs. 5.5 × 10^9^/L, *p* < 0.001); Lymphocyte count (1.1 × 10^9^/L vs. 1.4 × 10^9^/L, *p* = 0.01); Monocyte count (0.25 × 10^9^/L vs. 0.5 × 10^9^/L, *p* = 0.004); Platelet count (PLT, 95 × 10^9^/L vs. 165 × 10^9^/L, *p* < 0.001). Red cell distribution width (RDW) and serum albumin were similar. Serum ferritin was higher in Neutp patients (517 vs. 149 ng/mL, *p* = 0.002). BM cellularity, cytogenetic subgroups (data not shown), and the prevalence of megakaryocytic dysplasia were similar between both groups. Erythroid dysplasia was less common in Neutp than in Non-Neutp patients (50% vs. 72%, *p* = 0.04), while myeloid dysplasia (48% vs. 26%, *p* = 0.038) was significantly more common. There was no difference between the groups in the percentage of MDS patients with multi-lineage dysplasia (50% vs. 52%, *p* = 0.51). The median blast percentage (2% vs. 0%, *p* < 0.001) was higher in Neutp than in Non-Neutp patients but none of the patients had leukemic range blasts with counts exceeding 20%. Of the Neutp patients, 60.0% were classified as IPSS-R higher risk (score ≥ 3.5) at presentation, compared with Non-Neutp patients, where only 12.2% were classified as such (*p* < 0.001).

**TABLE 2:** Laboratory data for neutropenic and non-neutropenic MDS patients.

		All MDS *n* = 100	Neutp (*n* = 50)	Non-Neutp (*n* = 50)	*P*-value
**Median (IQR)**					
CBC	Hb (g/dL)	10.30 (8.57, 11.72)	9.65 (8.05, 11.28)	10.60 (9.10, 12.43)	0.015
	MCV (Fl)	92.00 (86.00, 101.00)	89.50 (86.00, 99.00)	96.50 (86.67, 102.00)	0.157
	RDW	16.00 (14.50, 18.70)	16.00 (14.00, 19.00)	16.00 (14.67, 17.55)	0.559
	WBC ×10^9^/L	3.70 (2.60, 5.53)	2.60 (2.10, 3.30)	5.55 (4.58, 8.20)	< 0.001
	Lymphocytes ×10^9^/L	1.20 (0.90, 1.72)	1.15 (0.83, 1.40)	1.40 (1.00, 2.38)	0.010
	Monocytes ×10^9^/L	0.40 (0.20, 0.70)	0.25 (0.10, 0.50)	0.50 (0.30, 0.78)	0.004
	PLT ×10^9^/L	134.50 (71.25, 212.75)	95.50 (53.75, 168.00)	165.00 (125.50, 237.50)	< 0.001
	Albumin (g/L)	40.00 (34.75, 42.00)	40.00 (36.50, 42.50)	40.00 (34.00, 42.00)	0.508
	Ferritin (ng/mL)	253.00 (95.50, 589.00)	517.50 (226.50, 1,078.25)	149.00 (56.25, 312.75)	0.002
BM Morpho	Cellularity% (mean ± SD)	41 (0.22)	42 (0.22)	40 (0.22)	0.633
	Erythroid dysplasia	61 (61%)	25 (50%)	36 (72%)	0.040
	Myeloid dysplasia	37 (37%)	24 (48%)	13 (26%)	0.038
	Megakaryocytic dys.	56 (56%)	25 (50%)	31 (62%)	0.314
	Blasts% median (IQR)	0.0 (0.0, 4.0)	2.0 (0.0, 5.0)	0.0 (0.0, 0.0)	< 0.001
BM Cyto *n* (%)	Good	75 (79%)	35 (73%)	40 (85%)	0.232
	Intermediate	11 (11.6%)	6 (12.5%)	5 (10.6%)	
	Poor	9 (11.6%)	7 (14.6%)	2 (4.3%)	
IPSS-R	HR (≥ 3.5)	36 (36.4%)	30 (60%)	6 (12.2%)	< 0.001

Ferritin: data were available for only 28 (in the neutropenic group and 38 in the non-neutropenic group, respectively). BM, bone marrow; IQR, interquartile range (Q1, Q3); Neutp, neutropenic patients; Non-Neutp, non-neutropenic patients; CBC, complete blood count; Hb, hemoglobin; MCV, mean corpuscular volume; RDW, red cell distribution width; WBC, white blood cell count; BM, bone marrow; Morpho, morphology; Cyto, cytogenetics (3 categories, combining good-very good and poor-very poor); IPPS-R, international prognostic scoring system, revised.

The median follow-up period was 29.5 months (IQR: 13.25, 55.75; maximum: 124 months). During the follow up period (Table 3), Neutp MDS patients were treated with significantly more G-CSF (40% vs. 12%, *p* = 0.003) and red blood cell (RBC) transfusions (54% vs. 32%, *p* = 0.043), but not erythroid stimulating agents (ESAs, 48% vs. 43%, *p* = 0.69).

**TABLE 3:** MDS treatments.

Treatment	All MDS *n* = 100	Neutp (*n* = 50)	Non-Neutp (*n* = 50)	*P*-value
G-CSF	26 (26%)	20 (40%)	6 (12%)	0.003
RBC transfusions	43 (43%)	27 (54%)	16 (32%)	0.043
ESA	45 (45.5%)	24 (48%)	21 (43%)	0.688

G-CSF, granulocyte colony stimulating factor; RBC, red blood cell; ESA, erythropoiesis stimulating agents.

**FIGURE 1:**
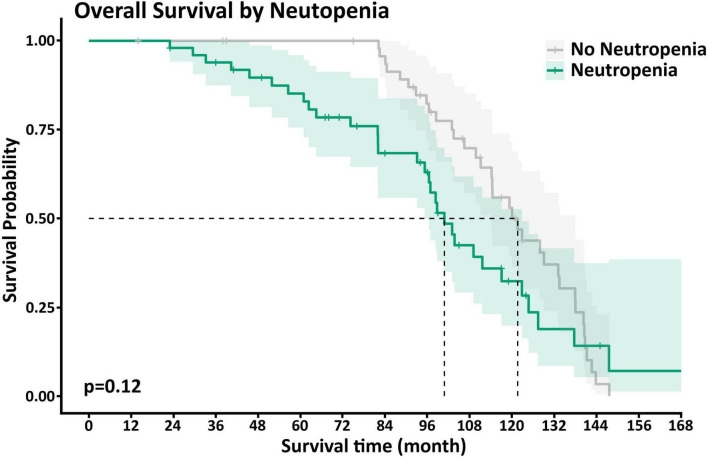
Kaplan–Meyer curves of survival for patients with (green) and without (gray) neutropenia. The median overall survival of neutropenic (Neutp) patients was shorter though without statistical significance (101 vs. 122 months, *p* = 0.12).

**FIGURE 2:**
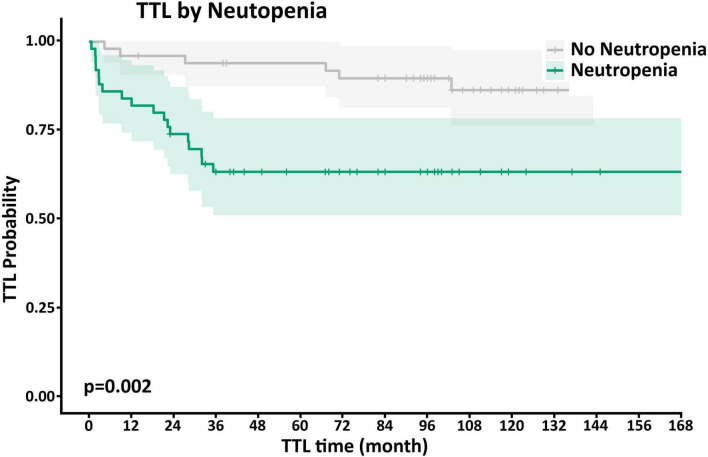
Kaplan–Meyer curves of leukemic transformation for patients with (green) and without (gray) neutropenia. During the follow-up period, 18 (36%) vs. 6 (12%) transformed to acute leukemia, respectively (*p* = 0.002). Note that neither group reached 50% transformation during the follow-up period. Therefore, the median leukemic transformation is not reported.

**FIGURE 3:**
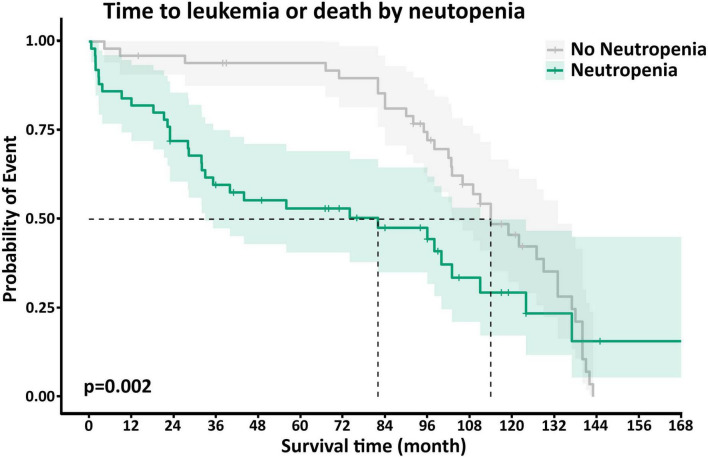
Kaplan–Meyer curves of the composite outcome of survival or leukemic transformation (whichever comes first) for patients with (green) and without (gray) neutropenia. The median was 82 months in the neutropenic (Neutp) group and 114 months in the Non-Neutp group (*p* = 0.035).

Neutp MDS patients experienced worse disease outcomes compared with Non-Neutp patients (Table 4): The median overall survival (OS) of Neutp patients was shorter (101 vs. 122 months, *p* = 0.12, see Kaplan–Meyer curves, Figure 1). During the follow-up period, 18 (36%) vs. 6 (12%) transformed to acute leukemia (*p* = 0.002). The time to leukemic transformation (TTL) is shown for both groups in Figure 2 and the difference is significant (*p* = 0.002). The median leukemic transformation rate is not reported because neither group reached 50% transformation during the follow-up period. Looking at the composite of time either to death or to leukemic transformation (whichever comes first), the median was 82 months in the Neutp group and 114 months in the Non-Neutp group (*p* = 0.035, Figure 3).

**TABLE 4:** Outcomes–survival, leukemic transformation and the composite endpoint.

	All MDS *n* = 100	Neutp (*n* = 50)	Non-Neutp (*n* = 50)	*P*-value
Median OS months [95% CI]	114 [103–123]	101 [95.3–123]	122 [114.2–138]	0.12
Leukemic transformation *n* (%)	24 (24%)	18 (36%)	6 (12%)	0.002
Composite, death or leukemic transformation months [95% CI]	103 [96–119]	82 [33–111]	114 [103–133]	0.035

Table 5 presents the results of the Cox proportional hazard models for mortality, leukemic transformation and the composite of both (whichever comes first). In these models, we included age, sex, the existence of CVD, cytogenetics (favorable vs. poor/intermediate) and neutropenia. In the model for mortality (Table 5 and Supplementary Figure 1), CVD at presentation was a significant risk factor for death (*p* = 0.034), while the role of neutropenia in mortality was of borderline risk (*p* = 0.056). For leukemic transformation, (Table 5 and Supplementary Figure 2), the cytogenetic profile (*p* = 0.031) and neutropenia (*p* = 0.03) were found to be significant.

**TABLE 5:** Cox proportional hazard models for mortality, leukemic transformation, and the composite of both (whichever comes first).

Variable	Mortality		Leukemic transformation		Composite outcome	
	HR [95% CI]	*P*	HR [95% CI]	*P*	HR [95% CI]	*P*
Age	1.0 [0.99–1.1]	0.154	0.99 [0.93–1.1]	0.87	1.0 [0.98–1.0]	0.67
Sex (male)	1.7 [0.96–2.9]	0.07	1.46 [0.54–3.9]	0.46	1.9 [1.10–3.4]	0.022
CVD	1.9 [1.05–3.3]	0.034	1.79 [0.58–5.6]	0.315	1.7 [1.0–3.1]	0.052
Cytogenetics (Poor)	1.8 [0.98–3.2]	0.06	3.05 [1.10–8.4]	0.031	2.4 [1.41–4.1]	0.001
Neutropenia	1.7 [0.96–2.8]	0.056	4.57 [1.16–18.0]	0.03	2.0 [1.2–3.5]	0.008

CI, confidence interval; Composite outcome, death or leukemic transformation (whichever comes first); CVD, cardiovascular disease.

In the composite model (mortality and leukemic transformation, whichever came first; Table 5 and Supplementary Figure 3), both cytogenetics and neutropenia were significant risk factors (*p* = 0.001 and 0.008, respectively), as well as gender (*p* = 0.022), while CVD was borderline (*p* = 0.052).

We then examined the impact of other blood indices and BM blasts on these outcomes using Cox regression. For mortality, the hazard ratios of monocytes, lymphocytes and platelets were not significant (1.1, 1.0, and 1.0, respectively). The hazard ratio of the BM blast count was 1.1 (95% CI 1.0–1.2, *p* = 0.044). Most interestingly, the effect of either neutropenia or anemia alone was not significant. However, the combination of both neutropenia and anemia was strong. This was true for mortality (HR [95% CI] 2.8 [1.47–5.2], *p* = 0.002), leukemic transformation (8.71 [3.03–24.8], *p* < 0.001), and the composite outcome of both (4.15 [2.25–7.7], *p* < 0.001). Table 6 summarizes these results, and the Supplementary Figures 4, 5, 6 present the detailed Cox regression data.

**TABLE 6:** New Cox proportional hazard models for mortality, leukemic transformation, and the composite of both (whichever comes first).

Variable	Mortality		Leukemic transformation		Composite outcome	
	HR [95% CI]	*P*	HR [95% CI]	*P*	HR [95% CI]	*P*
Age	1.0 [0.99–1.1]	0.203	0.94 [0.90–0.98]	0.006	1.0 [0.98–1.0]	0.755
Sex (male)	1.7 [0.97–2.8]	0.064	1.02 [0.42–2.45]	0.969	1.6 [0.94–2.8]	0.086
Neutropenia and Anemia	2.8 [1.47–5.2]	0.002	8.71 [3.03–24.8]	< 0.001	4.15 [2.25–7.7]	< 0.001
Blast%	1.1 [1.0–1.2]	0.044	1.2 [1.05–1.35]	0.003	1.1 [1.02–1.2]	0.021

CI, confidence interval; Composite outcome, death or leukemic transformation (whichever comes first). In this model, the combination of anemia and neutropenia is presented. The bone marrow blast percentage also had a significant, albeit smaller, impact.

**FIGURE 4:**
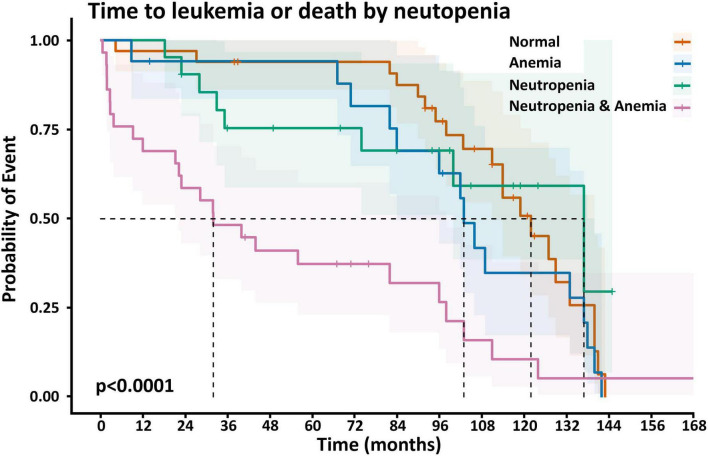
Kaplan–Meyer curves of the composite outcome of survival or leukemic transformation (whichever comes first) for patients with status of neither neutropenia nor anemia (orange), neutropenia alone (green), anemia alone (blue) and both neutropenia and anemia (purple). *P* < 0.0001.

Figure 4 demonstrates the Kaplan–Meyer curves for anemia and neutropenia, showing that neutropenia or anemia alone have only a small impact, while the combination of the two have a profound impact on the composite of mortality and leukemic transformation.

## Discussion

Neutrophils are an essential component of the myeloid compartment with anti-microbial and other immunogenic effects (15, 19). Their role in preventing or treating various inflammatory or other diseases, including infections, cancer and cardiovascular (CVD) has long been recognized (15, 20, 21). Decreased neutrophil numbers, neutropenia, as expected, has been associated with increased incidence of various infections (1, 6, 7, 15).

Neutropenia is a typical common abnormality in MDS (1, 3, 6, 7, 17, 18). While the role of neutropenia as a poor prognostic factor has been well recognized, a clear threshold has not been established.

The International Prognostic Scoring System Revised (IPSS-R) addressed the levels of counts (11). In IPSS-R, ANC < 0.8 × 10^9^/L added 0.5 points to the score. This was based on several previous studies. The Spanish group investigated the factors affecting prognosis and found that along with other parameters, neutropenia, especially severe (< 0.5 × 10^9^/L), was associated with shorter survival and higher rate of leukemic transformation (22, 23). They also reported that ANC < 0.8 × 10^9^/L was associated with higher potential infectious risk rather than that of 1.8 × 10^9^/L. The prognostic role of neutropenia was confirmed by others (24, 25).

While the presence of neutropenia as a poor prognostic marker has been established, the significant depth or severity of neutropenia as a prognostic adverse factor has not been elucidated. The few studies in the literature have focused on various ANC thresholds as a risk factor from severe (0.5 × 10^9^/L) (22), to intermediate (< 0.8 × 10^9^/L) (11), or mild (< 1 × 10^9^/L) (24). No clear evidence is available regarding milder neutropenia (< 1.5 × 10^9^/L). Moreover, in other studies ANC depth lacked additive prognostic value to the IPSS-R regarding survival or leukemic evolution (26). Thus, the question of the significant or threshold value to be prognostically important marker remains open, and this was the main goal of this study.

Today, there is a newer prognostic score which includes molecular, genetic information: IPSS-M. For this study genetic information was not uniformly collected on all patients, and we chose to exclude it. Genetic information is not universally obtained from all patients around the world, and this prompted us to perform this study without such information. It is important to establish the prognostic power of more readily available data such as mild neutropenia, especially for those for whom more advanced data are lacking.

The mechanism responsible for worse prognosis in neutropenic MDS patients remains unclear. It is likely that neutropenia induces susceptibility to infections. Indeed, infections are a common clinical manifestation and complication of MDS, which contributes to morbidity and excess mortality (16, 24, 27–32). We, with the European MDS group reported that 18% of mortality in MDS patients can be related to infections (30). Others reported on 64% infections as the cause of death (24). Infections are more common in treated MDS patients, especially with hypomethylating agents (24, 28, 33, 34). The common infections are bacterial pneumonias, skin abscesses, urinary tract infections and sepsis, while fungal and viral infections are less common (16, 24, 28, 31, 32). However, it is important to note that the determination of infection as the cause of death is somewhat difficult, considering the multiple potential problems in this patient population. For example, a patient with HR-MDS who transforms to AML develops pneumonia and eventually bleeds and dies, what is the cause of death? Often, the cause is decided by the attending physician and might be arbitrary. Unfortunately, in this study we could not address the issue of infections and focused on neutropenia and prognosis.

While neutropenia is likely to be a major predisposing factor for infections in MDS, several other immune defects have been reported, including impaired neutrophil function, B-, T- and NK-cell defects (16, 35–38). Interestingly, no correlation was found between ANC level and defective neutrophil function (36).

Other possible contributing causes for infections can be the consequences of iron overload due to RBC transfusions, the advanced age of most patients and their frequent comorbidities (16). Unfortunately, neither Filgrastim (G-CSF) administration in an attempt to raise ANC (39), or prophylactic antibiotics (16, 40) have succeeded in protecting against infections and/or improving prognosis.

Our study was designed with an attempt to answer clinically important questions regarding the characteristics and prognostic role of neutropenia at diagnosis in MDS patients, focusing on mild neutropenia (ANC < 1.5 × 10^9^/L). Comparing data from 50 neutropenic (Neutp) to 50 non-neutropenic (Non-Neutp) MDS patents, we found that hematologic parameters other than ANC, most known as prognostic markers (Hb, WBC, lymphocyte, monocyte and platelet counts), were significantly lower in the Neutp than in Non-Neutp patients. Serum ferritin was significantly higher in Neutp than in Non-Neutp MDS patients, probably reflecting the inflammatory nature. BM of Neutp MDS patients demonstrated more myeloid dysplasia and higher percentage of blasts compared with Non-Neutp patients, suggesting a higher degree of BM failure and more advanced disease. These features can be detected at disease presentation.

Indeed, the outcomes of Neutp patients in our study were significantly worse: More Neutp than Non-Neutp patients transformed to acute leukemia (36% vs. 12%), but given the relatively short follow up neither group reached the 50% to determine median transformation time. The median overall survival was shorter (101 vs. 122 months), with no statistical significance, perhaps due to the small numbers.

What is particularly interesting in this study is the impact of neutropenia together with anemia. The impact of even mild neutropenia is important, but may not take into account whether other indices are also low. We found that other white cell indices, namely lymphocytes and monocytes had no significant impact. Looking at the other cell lines, platelets had no effect, but anemia did. When examining this in greater depth, we found that either neutropenia or anemia alone had a minimal impact with no statistical significance in this study. The combination of the two, however, had a profound impact on both mortality and leukemic transformation.

Blast percentage was also seen to be important for prognosis in MDS. This, however, is a bone marrow index. What is important in this study is that the readily available peripheral blood indices also reflect the severity of disease and are important for disease prognosis.

MDS is a disease of the elderly, and this patient population often suffers from comorbidities, especially CVD. Thus, we studied the prognostic role of 5 variables (age, sex, CVD, cytogenetics and neutropenia) as risk factors for mortality, leukemic transformation and composite outcome (both, whichever comes first) in a Cox proportional hazard model. While CVD was important risk factor for mortality (neutropenia borderline), both neutropenia and poor cytogenetics were risk factors for leukemic transformation and the composite outcome.

As expected, it is likely that patients with neutropenia upon diagnosis already have more advanced disease. This report supports the use of mild neutropenia as an independent prognostic feature.

The study suffers from other limitations. The major one, as mentioned above, is the lack of information regarding infections. Also, the retrospective nature of the study and using single institute data are inherent problems in interpreting such studies. The small numbers of patients studied did not allow us to distinguish between milder and more severe neutropenia, suggesting that caution is needed in drawing conclusions. Finally, we could not evaluate the role of G-CSF and whether increasing the neutrophil count might improve the prognosis.

Nevertheless, the study shows that even without addressing the incidence and nature of infections, neutropenia, even mild, is a prognostic factor in MDS. It is also readily available. Whether the prognostic role of neutropenia is due to infections or other mechanisms, remains an open question. Our findings call for a consideration of neutropenia as a prognostic marker, probably more than previously considered.

In conclusion, we showed that mild neutropenia (< 1.5 × 10^9^/L) is a poor prognostic factor in MDS. These patients are more anemic and thrombocytopenic, their BM demonstrates evidence of more advanced failure, their leukemic transformation is higher, and the overall survival might be shorter than of non-neutropenic MDS patients. This is especially true if anemia is also present. Future studies with larger groups of patients will clarify whether the neutropenic effect is associated with infection, BM failure or other mechanisms.

## Data Availability

The raw data supporting the conclusions of this article will be made available by the authors, without undue reservation.
