# Supplementary material for: Neutropenia (even mild) and anemia are poor prognostic factors in myelodysplastic syndromes
Source: Front Med (Lausanne). 2025 Jul 11;12:1558585. doi: 10.3389/fmed.2025.1558585 (PMC12289705; doi:10.3389/fmed.2025.1558585)
Supplement: Supplementary file 1 [file Supplementary_file_1.docx]

**Neutropenia (even mild) and Anemia are Poor Prognostic Factors in Myelodysplastic Syndromes**

**Supplementary figures:**

**Figure 1S: Original model. Neutropenia has borderline effect on mortality.**


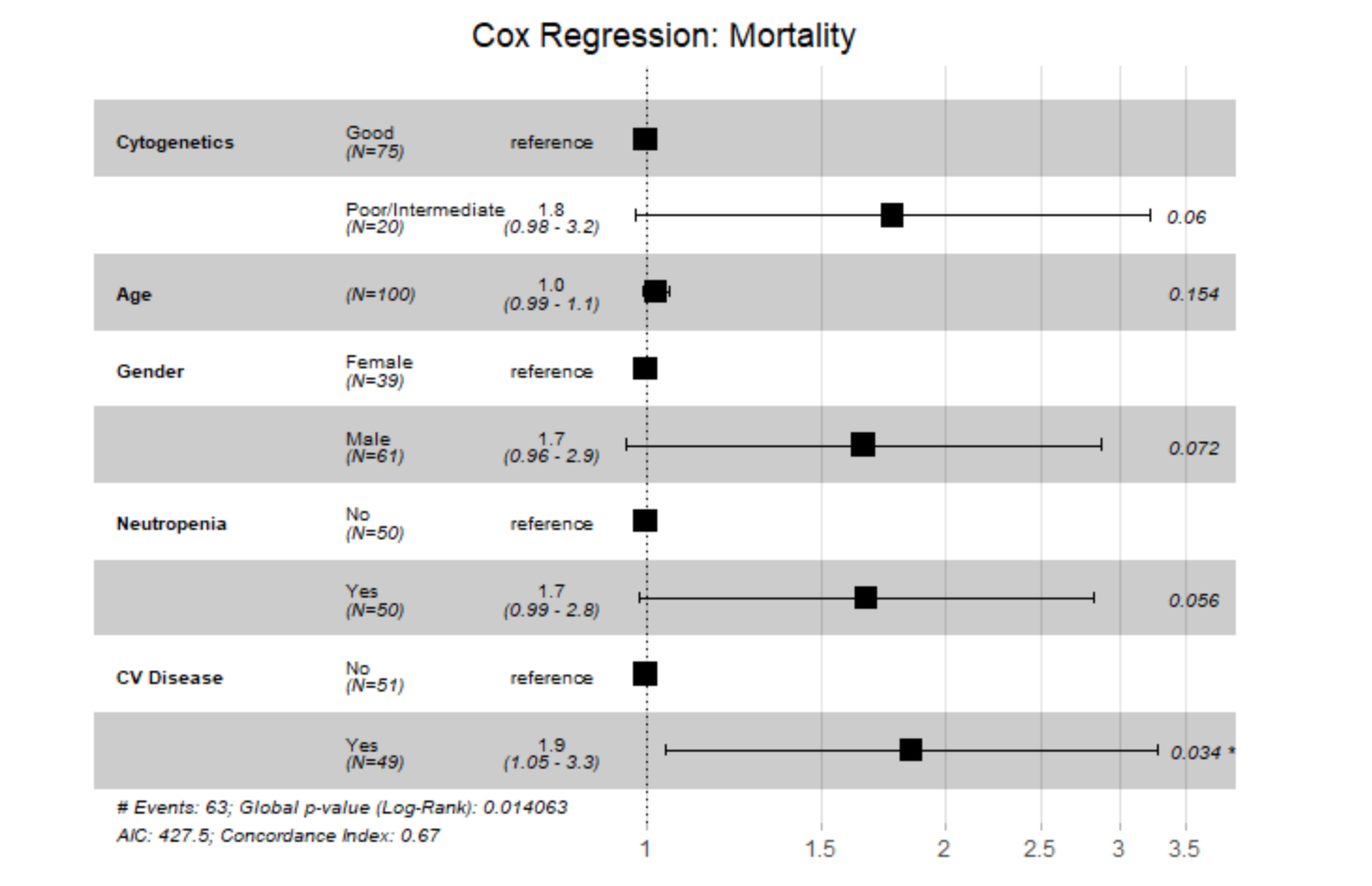


**Figure 2S. Original model. Neutropenia has impact on leukemic transformation**


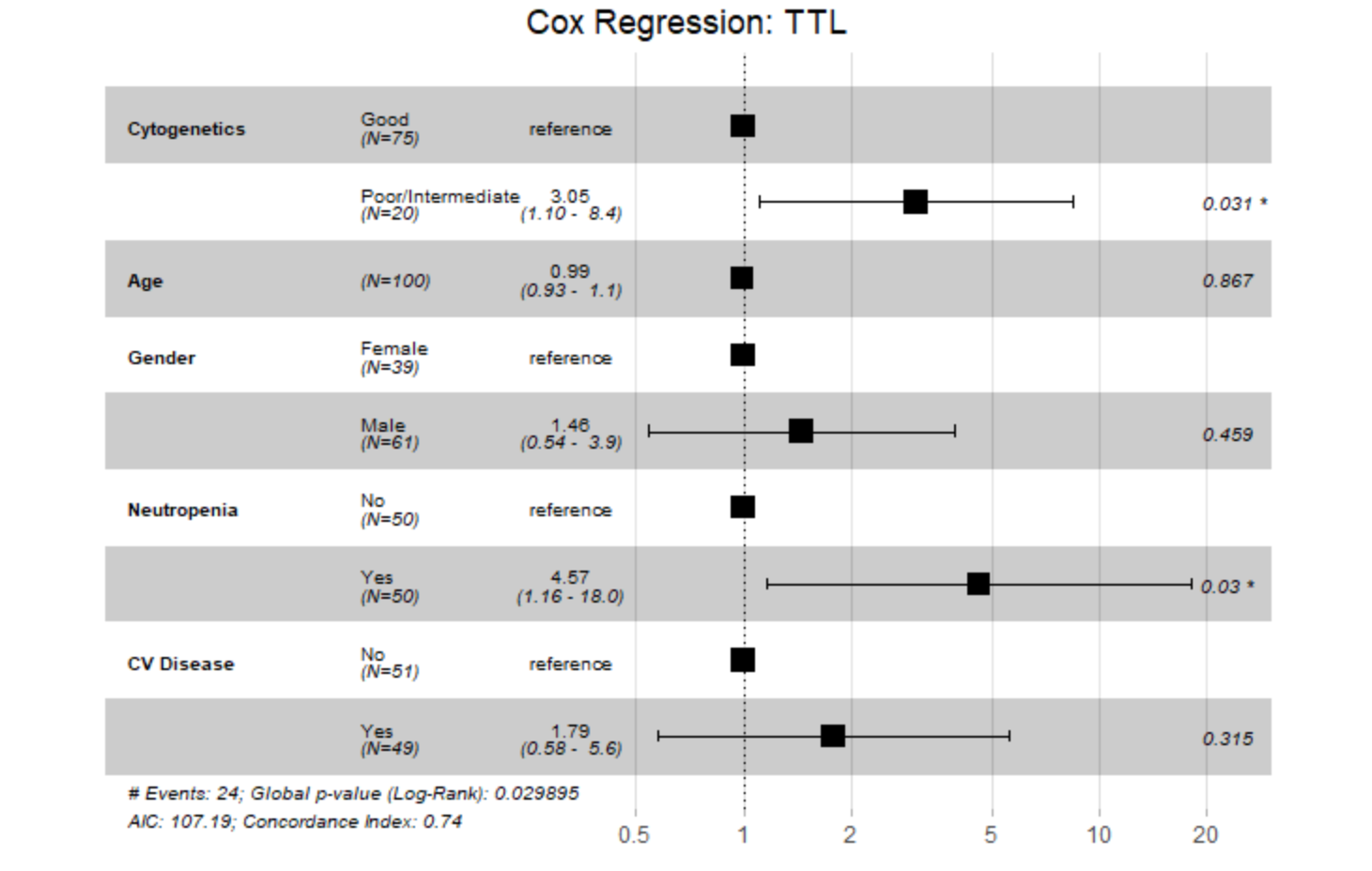


**Figure 3S. Original model. Neutropenia has impact on composite outcome of mortality and leukemic transformation.**
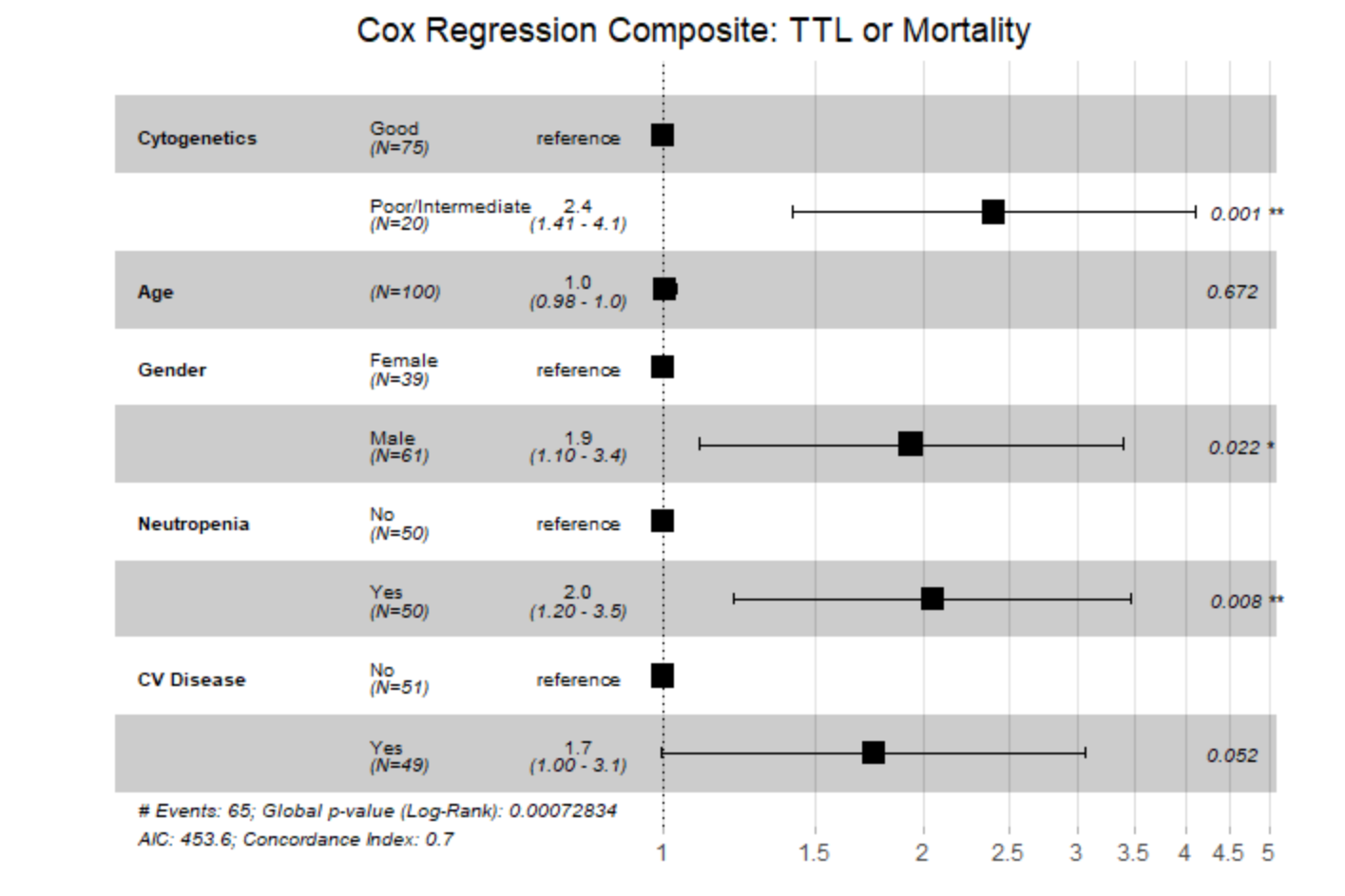


**Figure 4S. Model with the addition of monocytes, lymphocytes, platelets, blasts and the combination of neutropenia and anemia. The combination of neutropenia and anemia has a strong impact on mortality.**

**
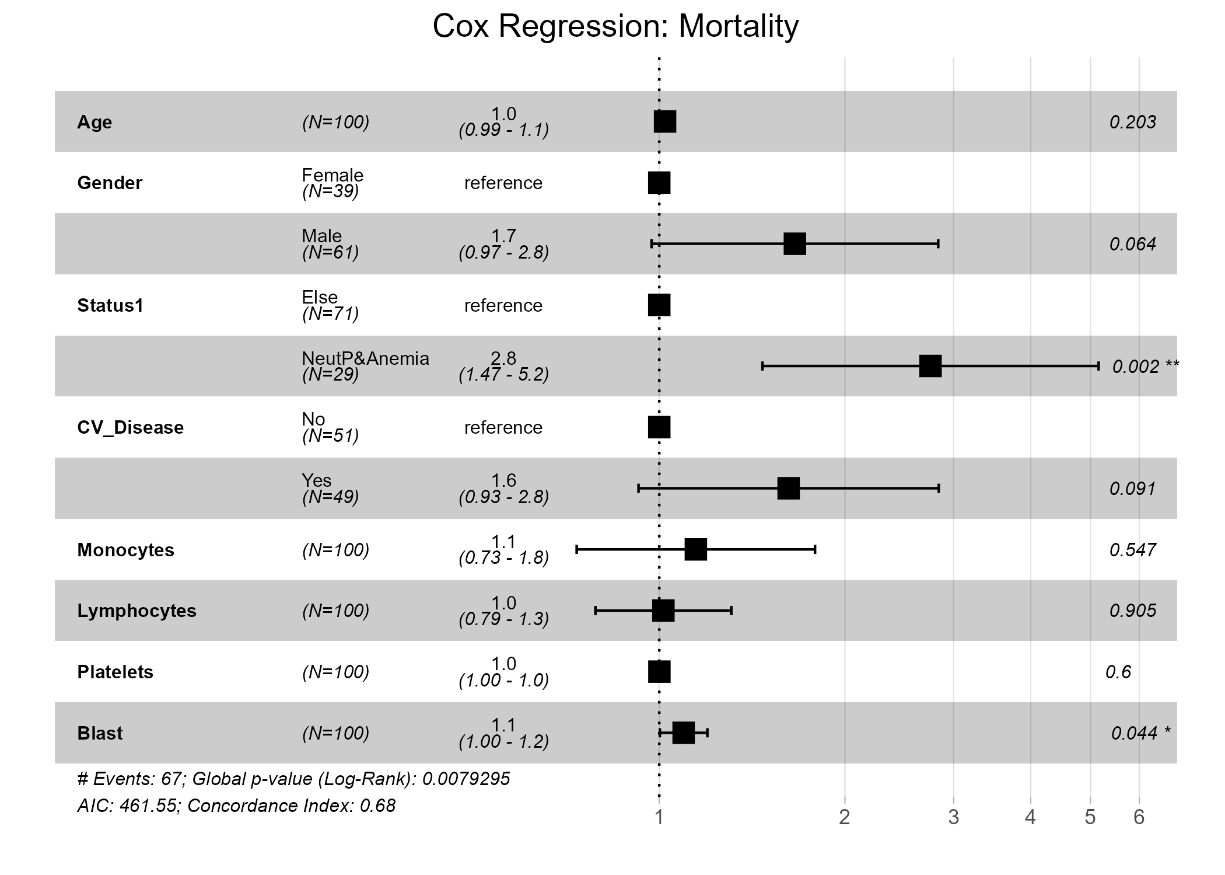
**

**Figure 5S: Model with the addition of monocytes, lymphocytes, platelets, blasts and the combination of neutropenia and anemia. The combination of neutropenia and anemia has a strong impact on leukemic transformation.**

**
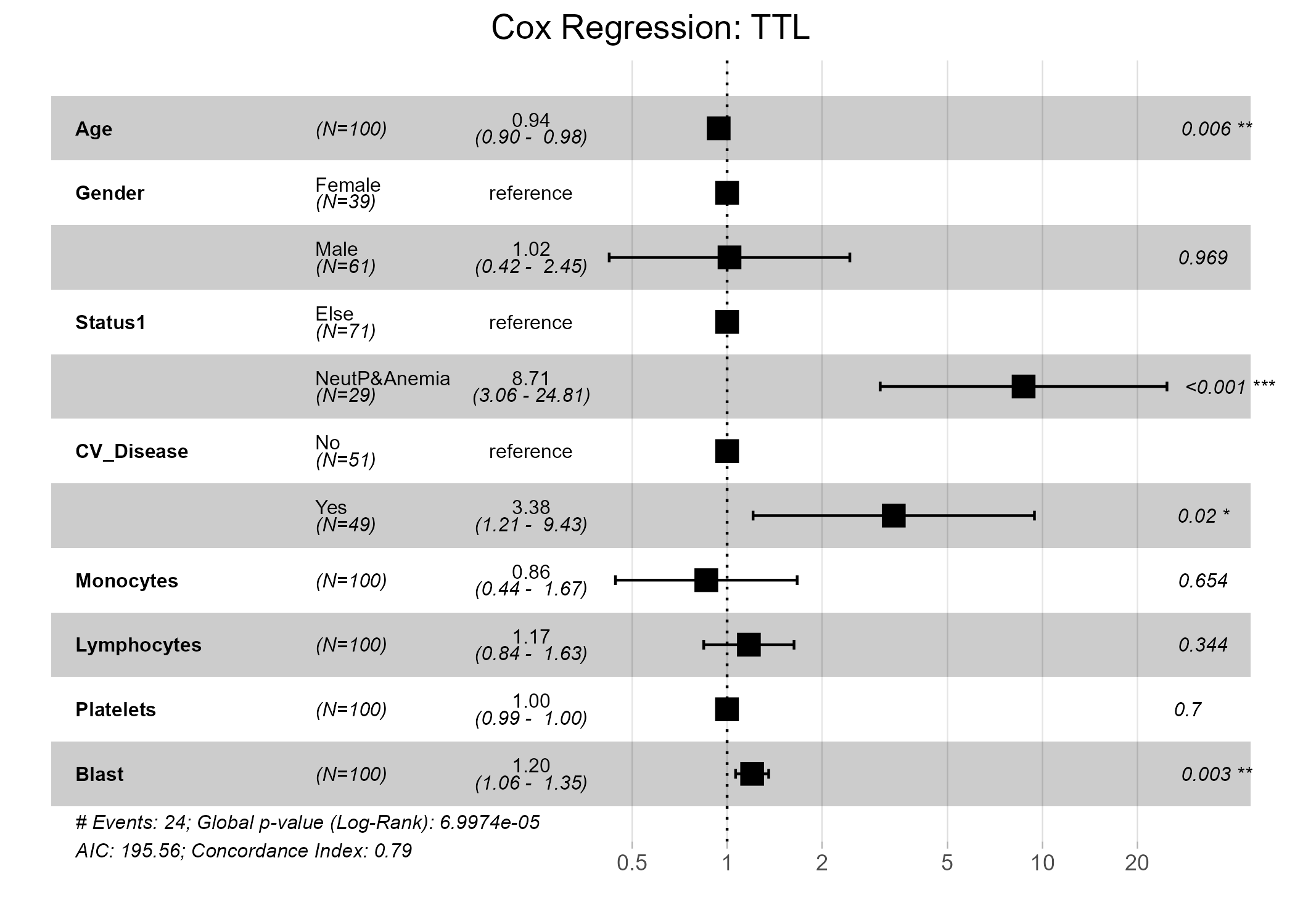
**

**Figure 6S: Model with the addition of monocytes, lymphocytes, platelets, blasts and the combination of neutropenia and anemia. The combination of neutropenia and anemia has a strong impact on the composite outcome of mortality and leukemic transformation.**

**
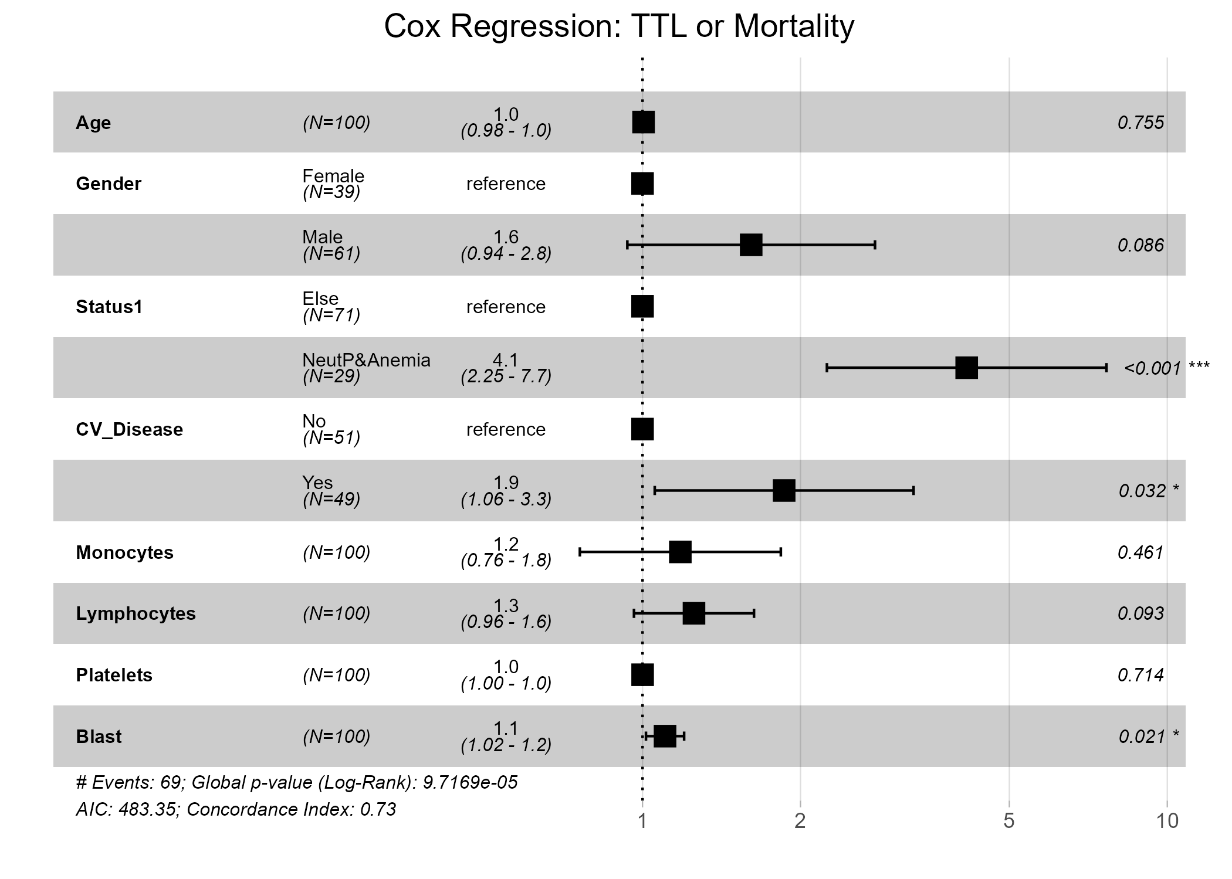
**
